# Academy of Medicine and Surgery of Kentucky

**Published:** 1881-02

**Authors:** 


### ACADEMY OF MEDICINE AND SURGERY OF KENTUCKY.

---

*Ophthalmic Pathology.*—Under the call for "presentation of pathological specimens," Dr. D. S. Reynolds exhibited several eyes, and, in presenting them, made the following remarks:

There is an interesting history connected with these specimens. This is the skeleton remains of an eye the vision of which had been lost many years ago, from a neglected inflammation of the iris. It is plainly perceptible, from the mass before you, that the globe must have been not less than two inches in diameter. About five years ago the last perception of light disappeared. The unfortunate subject from which this specimen was taken was about thirty-eight years of age, and had been living in a malarious district in the southern portion of this State. He suffered from an inflammation of the iris, which, as I remarked before, was neglected, until finally it involved the ciliary body and extended its ravages to the choroid. Dropsical effusion then took place, distending the globe so much as to make serious pressure upon the structures between it and the orbit and to produce such irritant effects upon the brain as to cause epilepsy, for which he had been ineffectually treated by prominent physicians and quacks in the cities of St. Louis, Cincinnati, Philadelphia and New York, within the last five years. He had been given nitrate of silver, bromide of potassium, atropia, nitrite of amyl, and all kinds of treatment known to the profession.

In the last two or three months previous to his visit to this city, he had suffered as many as three attacks per month; and probably on account of the excitement induced by the thought of an operation, after his arrival here had two attacks in twenty hours.

I enucleated the eye which I here present, and he has not had a particle of headache since. It is now three weeks since the operation was performed, and up to this time he has had no return of the epileptic seizures.

He is a man well nourished and of great muscular power, and had never been sick until about eight years ago, when he had what he considers an attack of neuralgia in the right eye. This was followed by the slowly collecting effusion, the pain becoming more of an aching character, often extending from the temple to the occiput. Five years ago he had an attack in the left eye, of a similar character, and shortly after that he lost the sight in this eye. I report this case to show that although the amount of pain and distention was something unusual, the nervous system was so influenced as to cause epilepsy. It is not common to see the ball so distended as to be almost immovable. In this case it was necessary for me to cut into the distended walls of the globe before the muscular and connective tissue attachments surrounding the ball could be severed. There was not sufficient room to seize the muscles between the distended globe and the orbit. The remaining eye is quite free from irritation, and in consequence of that fact, it was allowed to remain.

*Specimen* 2.—Here is an eye that was taken from a young lady two months ago. The eye had been injured in infancy, by a puncture with a pair of scissors. The remarkable feature of the case is that, although the puncture was made through the sclero-corneal juncture and ciliary body, and the eye shrunken to a mere a stump, the young lady had gone to school and obtained an education, and had never suffered any inconvenience nor discomfort in the use of the fellow eye.

Here was a puncture inflicted when she was three years of age, and she is now nineteen, the shrunken globe remaining all this time without causing serious pain or other morbid manifestation in the opposite eye. It is almost universally believed by specialists that an eye destroyed by a punctured wound of the ciliary body is certain, if allowed to remain, to set what is known as sympathetic irritation in the fellow eye, which will lead to fatal results, so far as the vision is concerned. Mr. G. Lawson, in the reports of the Royal London Ophthalmic Hospital for 1872 or '73, records the case of man who had lost an eye from injury; the globe underwent atrophic changes, such as are shown in this specimen, and the man had gone on about forty-two years without suffering any inconvenience in the other eye, when suddenly a sympathetic irritation became manifest in the opposite eye, which soon began to grow dim, and in a few weeks he was admitted to the hospital, with bare perception of light. Though the lost eye was, on his admission to the hospital, removed, the opposite one went on to complete blindness, and Mr. Lawson was sure the one eye could have been saved by the timely removal of the blind eye.

*Specimen* 3.—This is the eye of a gentleman only twenty-eight years of age; it was destroyed by the kick of a mule, eight months before he came to have it removed. The calk of the shoe worn by the mule struck the man on temporal margin of the cornea, and ruptured it. An intelligent gentleman, who was somewhat acquainted with the structure of the eye, told him there was a rent just within the cornea, on the temporal side. In this specimen, as you see, the whole cornea has been removed by absorption, and the atrophic changes which followed by continuity of structure, producing contraction of the whole globe. In September the eye became tender and painful, but it seemed to get well again, and about the middle of October it was again effected, and this time the sound eye grew sensitive and watery, which led him to consult a physician, who told him to consult a specialist. This attack subsided, he waited another week, when he was again attacked, and with corresponding dimness and morbid sensibility to light in the fellow eye.

When he came I removed the eye, althongh there was considerable irritation at the time. The other eye remained a little sensitive to light, and was somewhat dim for two weeks. After that time a test of the vision revealed about the normal acuity. About six weeks after the removal of the eye he came back to get an artificial eye; and, though the structures seemed to have regained their normal condition, all signs of irritation having disappeared, the instant an artificial eye was introduced he suffered pain, and lachrymation of the other eye. I advised him to return home for the present and return in two months.

*Specimen* 4.—This eye has a much sadder history connected with it. The young gentleman to whom it belonged had the misfortune to contract a gonorrhœa on the 4th of July. About the middle of the month he discovered he had something the matter, but it did not occur to him at the time that it was due to the exposure on the 4th, and he paid little attention to it. He was spending his summer vacation in a general frolic, and came here to Louisville to look around. While here he came to my office to deliver a message from a physician in the neighborhood where he lives. He told me he believed he had gonorrhœa. I sent him to a medical friend and soon had forgotten all about him. About ten days afterwards his physician called and told me he believed the young gentleman had lost one of his eyes, and he desired me to go and see him. I found the right cornea had sloughed out *en masse*, and the left was becoming hazy. Careful attention succeeded in saving the left eye, but the right affords the specimen before you. This shows the importance of early attention to gonorrheal ophthalmia. There is an important lesson to be derived from a study of these cases. They show, in the first place, that though an eye be absolutely blind, it may give rise to so much irritation as to cause epilepsy in a strong and vigorous man. Another specimen of an eye destroyed in a manner most likely to create serious trouble, is followed by no inconvenience or discomfort in the use of the opposite eye. Here is another case with only the cornea lacerated, no other part damaged, that gets well and in a few weeks after recovery he begins to suffer recurrent attacks of inflammation in it, which causes sensibility to light in the opposite eye, and the patient is threatened with a sympathetic ophthalmia.

Another, a young man in good health, contracts a gonorrhœal inflammation, and, failing to give prompt attention to it, remains in the obscurity of a boarding house until the eye sloughs nearly out, and the remainder must be removed.

To morrow will be three weeks since three gentlemen, on the Ash Creek Road, were out shooting birds. While they were considerably separated, a covey rose, and the gentleman at the extreme left fired toward the right, and a shot struck one of his companions in the right eye, passing through the cartilaginous portion of the upper lid, directly in the vertical meridian. He came to see me in less than an hour after the receipt of the injury. The anterior chamber was filled with blood, and there was a large rupture at the point indicated, showing that the shot had penetrated the globe. I gave him a purgative dose of calomel, and ordered a solution of atropia and morphia, and directed him to bathe the eye with cold water, and under no circumstances to cover it up. In a few days the wound healed and the blood disappeared. At present a normal condition of the aqueous chambers is present, except where the shot came through the wall above. Careful examination shows no detachment of the retina; the red reflex from the fundus may be seen all round, and the shot must be very near the ciliary body in the anterior and inferior portion of the globe. He has had but little pain in it up to this time, and, strangely enough, there has been no inflammation of the damaged iris except at the point where the shot entered. He was able to count my fingers yesterday with that eye; and while I have no hope of saving the vision, I have some hope of the shot being encysted and keeping quiet. If it does not, however, I propose, with his consent, to perform optico ciliary neurotomy. It will probably save him what appears to be a good eye, which is much more convenient and better looking than any artificial eye can possibly be made.

*Croup, with Tracheotomy.*—Dr. F. C. Wilson: I may mention two interesting cases I have met with in the last five days. Last Sunday, I was called to see a little girl, eight years of age, who had been sick for two or three days, with what was thought to be croup. All the domestic remedies for such troubles had been tried, and when I saw her first she was breathing with great difficulty, both inspiration and expiration being labored. She had a muffled, croupy cough, and a temperature of 110½°, quick pulse and a tendency to cyanosis. I informed the parents at once that the child was very ill, as I felt satisfied that it was a case of membranous croup. I ordered an emetic of turpeth mineral, and for the night five grains of Dover's powders, also, as a local application, cold compresses, to be frequently changed. I saw her again next morning at eleven. She had freely vomited, and had thrown up one or two particles of organized material, along with a considerable quantity of very tough mucus, and she was relieved. That night she rested easier until about six A. M., when there was much more obstruction, and a good deal of cyanosis. I administered oxygen, which restored the red color to her lips, and I then suggested to the family the necessity for tracheotomy, which met with great opposition, as I knew it would, the family being German. The next forenoon I called Dr. Octerlony in consultation, and we agreed at once as to the diagnosis, and the only change in the treatment suggested was the addition of five grain doses of carbonate of potash every half hour. In the afternoon the obstruction had increased, the respiratory murmur was feeble on both sides of the chest, and a whistling murmur was present, from the obstruction. I again suggested tracheotomy. They still refused.

At 9 P.M. she was worse, and at 9.30 her father came and said if I thought the operation could offer any hope, they would consent. I thought the hope then a forlorn one, but I went at once, and, with the assistance of a druggist in the neighborhood and what few instruments I had at hand, and what I could improvise, I determined to open the trachea and insert a tube. I made an insertion through the skin, and was delayed a good deal by the capillary hemorrhage. Finally, the trachea was opened, the lips of the wound separated and the child breathed with comparative ease. I had suspicions, however, that the membrane extended below the point of incision, and these suspicions were confirmed by the introduction of the tube, which had to be immediately removed. I concluded to hold the edges apart, in order to let her breathe more easily, and I could distlnctly see the yellowish-white membrane obstructing the trachea below the point of incision; but at no time did she breathe with that quiet ease which is noticeable in cases where the membrane does not extend below the point of incision. In this case it was merely temporary relief. I held the edges apart for three or four hours, when the necessity caused me to improvise a speculum. which I made out of a hair-pin. This I found to answer every purpose, but the child died at half-past four the next morning.

That afternoon I was called to see a little German boy, five years old, sick two days in the same way. I found his fingers and lips blue, obstruction in both inspiration and expiration, and the same kind of muffled cough noticed in the other case. I explained to the father the great danger the child was in. After prescribing an emetic of turpeth mineral, I promised to see it again in the evening. Accordingly, at 8:30 I went out and found the child dying.

These were two fatal cases in the same day. I have met with twelve in all my experience, and of the twelve cases have succeeded in saving four. Two I saved, I believe, by the inhalation of oxygen gas, keeping the patient alive until the membrane was detached and expelled; in a third I attribute the relief to the inhalation of lactic acid, and in a fourth to the insufflation of pepsine, and inhalations of lime and bromide of potash and the carbolic acid spray, to which I attribute the most importance.—*Medical and Surgical Reporter.*

